# Ocular leptospirosis as the sole clinical manifestation in a pediatric patient: an atypical case

**DOI:** 10.22336/rjo.2026.41

**Published:** 2026

**Authors:** Marta Moro-Muniz, Javier Cañas Costa, Honorio Barranco González, Amparo Ortiz-Seller

**Affiliations:** 1 Department of Ophthalmology, Hospital de Manises, Valencia, Spain; 2 Department of Ophthalmology, Hospital Universitari i Politècnic La Fe, Valencia, Spain

**Keywords:** ocular leptospirosis, pediatric uveitis, papillitis, retinal vasculitis, immune-mediated inflammation

## Abstract

**Objective:**

To describe an atypical presentation of ocular leptospirosis in a pediatric patient without systemic symptoms, highlighting its diagnostic challenges and therapeutic approach.

**Case Presentation:**

A 9-year-old previously healthy girl presented with unilateral papillitis and vitritis, progressing to 360º retinal vasculitis as the sole manifestation of subclinical leptospirosis. Serology was positive for Leptospira spp. (IgM and IgG), although PCR testing in aqueous humor and urine was negative. The condition was interpreted as a post-infectious immune-mediated response. Treatment included systemic corticosteroids, intravenous ceftriaxone, and intravitreal triamcinolone, resulting in complete anatomical and visual recovery.

**Discussion:**

Ocular leptospirosis can appear as an isolated manifestation in the immune phase of infection, even in the absence of general symptoms or epidemiological evidence. Pediatric cases are rare and often underdiagnosed due to the negative microbiological tests in late stages. This case also highlights the importance of considering environmental exposures, such as flooding, as potential risk factors.

**Conclusion:**

This case underlines the need to include leptospirosis in the differential diagnosis of posterior uveitis and papillitis of unknown origin in children. Early multidisciplinary management and appropriate immunomodulatory treatment can prevent permanent visual sequelae.

## Introduction

Leptospirosis is a bacterial zoonosis caused by spirochetes of the genus *Leptospira*, with global distribution but predominance in tropical and subtropical regions, especially in contexts of heavy rainfall, flooding, or poor sanitary conditions. Transmission to humans usually occurs through direct contact with the urine of infected animals, typically rodents, or indirectly through contaminated water. Although traditionally associated with rural settings, outbreaks have also been documented in urban areas and developed countries, often linked to recreational water activities [1,2].

From a clinical perspective, leptospirosis presents with great variability, ranging from subclinical infections to severe forms with renal failure, pulmonary hemorrhages, or meningitis. In its classic form, it follows a biphasic course: an acute phase followed by an immune phase, during which systemic complications may arise, including ocular involvement [2,3].

Ophthalmological manifestations, although described over a century ago, remain underdiagnosed. In the acute phase, mild signs such as non-purulent conjunctivitis, episcleral hyperemia, or subconjunctival hemorrhages may be observed. However, in the immune-mediated phase — typically between the second and sixth week after infection — more severe forms may appear, such as anterior uveitis, panuveitis, retinitis, papillitis, retinal vasculitis, choroiditis, or even neuroretinitis [3-5]. These ocular manifestations may be the sole clinical expression of a subclinical leptospirosis, making recognition even more difficult. Diagnosis in these cases relies on a high index of clinical suspicion. It is usually supported by serology (IgM and IgG), whereas direct tests such as PCR in urine or aqueous humor tend to become negative in later phases [4].

Therefore, ocular leptospirosis represents a significant diagnostic challenge, especially outside of endemic areas. In pediatric age groups, its presentation is exceptional and poorly documented, warranting consideration in the differential diagnosis of posterior uveitis or retinal vasculitis of unknown cause, even in the absence of general symptoms. Treatment should be adapted to the disease stage: in the acute phase, antibiotic therapy (doxycycline, amoxicillin, or ceftriaxone) is recommended; in the immune-mediated phase, systemic corticosteroids are prioritized; and, in some cases, immunomodulatory therapies may be required [5,6].

We present the case of a previously healthy girl who developed unilateral papillitis and retinal vasculitis as the sole manifestation of subclinical leptospirosis, with an evolution compatible with an immune-mediated mechanism. This is an uncommon form of presentation, especially in childhood, whose early recognition is essential to prevent permanent visual sequelae. The patient lived in an area affected by the DANA that caused flooding in the Valencian Community in 2024, potentially increasing exposure to the pathogen.

## Case report

We present the case of a 9-year-old girl with no relevant personal or family history, fully vaccinated and without known allergies, who presented to the Pediatric Emergency Department in March 2025 with an intermittent headache for the past 4 days, localized to the right frontal and periocular regions. The headache was initially accompanied by whitish floaters, which later evolved into colored visual perceptions. She did not report photopsia, photophobia, or vision loss. Afebrile and in good general condition, she had normal physical and neurological examinations, except for tenderness to percussion over the frontal and maxillary sinuses. There were no signs of increased intracranial pressure or systemic involvement.

**Fig. 1:**
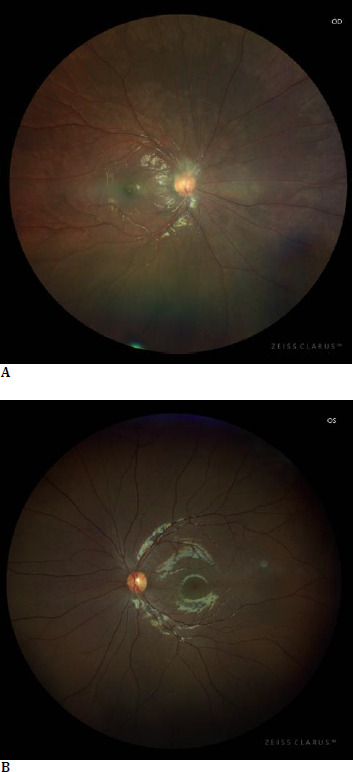
Color fundus photographs of both eyes. The right eye (RE) shows optic disc edema with poorly defined margins, peripapillary hemorrhages, vitritis, and signs of peripapillary vasculitis, including venous engorgement and vascular sheathing (A). The left eye (LE) presents a completely normal fundus, with no signs of inflammation or structural abnormalities (B)

Due to persistent symptoms, an ophthalmology consultation was requested. On initial examination, uncorrected visual acuity (VA) was 1.0 in both eyes, with a relative afferent pupillary defect (RAPD) in the right eye (RE), peripapillary hemorrhages, venous engorgement, and turbid vitreous observed on fundus photography and OCT (Fig. 1A, B). The fundus of the left eye (LE) was normal. Visual field testing revealed an inferior altitudinal defect in the RE.

Hospital admission was indicated to complete the work-up. In the context of unilateral papillitis, general blood tests, serologies, and an aqueous humor sample for PCR were requested. Serology was positive for *Toxoplasma gondii* and *Leptospira spp*. IgG, with positive IgM only for *Leptospira*. PCR tests in aqueous humor and urine for both agents were negative. Given the initial suspicion of infectious etiology, empirical treatment with cotrimoxazole and doxycycline was initiated.

**Fig. 2:**
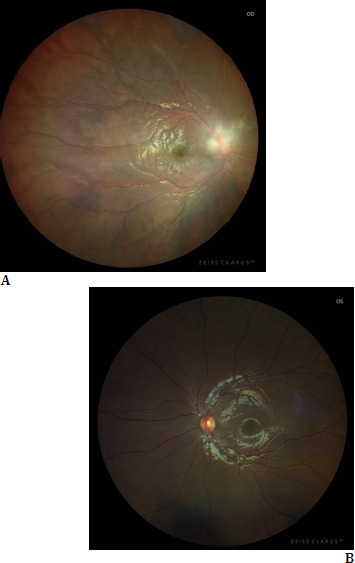
Color fundus photographs of both eyes. The right eye (RE) shows optic disc edema with poorly defined margins, peripapillary hemorrhages, vitritis, and signs of peripapillary vasculitis, including venous engorgement and vascular sheathing (A). The left eye (LE) presents a completely normal fundus, with no signs of inflammation or structural abnormalities (B)

**Fig. 3:**
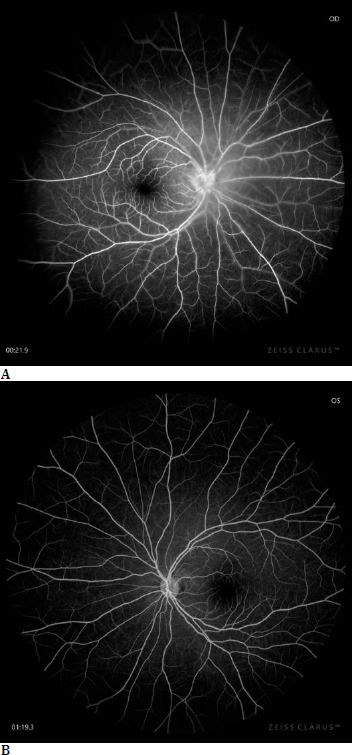
Fluorescein angiography of both eyes. In the right eye (RE), hyperfluorescence of the optic disc is observed with blurred margins and progressive dye leakage, along with vascular engorgement and signs of peripapillary vasculitis. No peripheral ischemic areas are observed at this stage (A). The left eye (LE) shows a completely normal angiography, with no perfusion abnormalities or signs of inflammation (B)

During follow-up, the patient experienced functional worsening with a decrease in visual acuity to 0.7 in the right eye (RE), development of grade 2+ vitritis, and progression of optic disc edema. Fundus photography revealed 360º retinal vasculitis with vascular sheathing, no exudates, and optic disc edema, with early signs of peripheral ischemia (Fig. 2A, B, 3A, B).

A multidisciplinary clinical meeting was held with the Ophthalmology, Pediatric Neurology, Rheumatology, and Infectious Diseases departments. The condition was interpreted as a post-infectious immune-mediated manifestation of leptospirosis, based on the compatible clinical context, positive IgM serology, and subacute evolution. Oral prednisone (0.5 mg/kg/day) was initiated for 6 weeks, followed by gradual tapering. Given the persistent inflammatory course and the extent of the condition, intravenous ceftriaxone 2 g every 12 hours was also administered for 7 days as a potential adjuvant, despite negative PCR results.

Subsequently, an intravitreal injection of triamcinolone acetonide was administered to the right eye (RE), resulting in significant improvement in retinal inflammation and papillitis. During follow-up, the patient developed secondary ocular hypertension (IOP up to 45 mmHg measured with iCare), which was controlled with combined topical therapy (Cosopt®, Monoprost®, Alphagan®).

At 10 weeks from symptom onset, the patient had an uncorrected visual acuity of 1.0 in both eyes, with good structural recovery on OCT and fundus photography. A slight diffuse atrophy of the ganglion cell layer persisted in the RE, with no active signs of inflammation or ischemic progression.

## Discussion

Ocular leptospirosis is an uncommon but potentially serious manifestation of a systemic infection that often presents subclinically or goes unnoticed during the acute phase. The case we presented—a previously healthy pediatric patient with unilateral papillitis that progressed to 360º retinal vasculitis—illustrated an atypical presentation in childhood, without a relevant infectious history or clear systemic symptoms, posing a significant diagnostic challenge.

Ophthalmological manifestations may be the only clinical expression of Leptospira spp. infection, especially during the immune-mediated phase, when the bacterial load is no longer detectable, and PCR testing in blood, urine, or aqueous humor yields negative results. In this phase, diagnosis relies primarily on the clinical context and serology, as in our patient, who had positive IgM and IgG with no active genomic detection [1,2].

An additional epidemiological detail of note is that the patient lived in an area affected by the DANA (a cut-off low weather event) that struck the Valencian Community in the fall of 2024. This climatic phenomenon caused torrential rains and flooding across large urban and rural areas, creating a favorable environment for exposure to Leptospira spp. through contaminated stagnant water—a well-documented transmission route in outbreaks following extreme weather events [1]. Although no systemic symptoms or clear exposure history were identified in the patient, this circumstance strengthens the plausibility of an infectious origin.

Clinically, the association of papillitis, vitritis, and retinal vasculitis in a young patient without systemic signs suggests an infectious or immune etiology. It is essential to conduct a thorough evaluation to rule out other entities with similar features, such as Behçet’s syndrome, Eales disease, or uveitis due to other infectious agents. While unilateral involvement can be seen in various causes of posterior uveitis, the presence of generalized peripheral vascular sheathing and a good response to corticosteroids suggests an immune-mediated mechanism. This pattern is consistent with findings in recent case series of ocular leptospirosis [3-5].

The optimal treatment for ocular leptospirosis has not yet been standardized. During active phases, antibiotics such as doxycycline or amoxicillin are widely accepted, although their role in the immune-mediated phase remains controversial. In such cases, systemic corticosteroids are the mainstay of therapy, and early administration has been associated with better functional and anatomical outcomes. In the case described, a combination of oral prednisone, intravenous ceftriaxone, and intravitreal triamcinolone helped control inflammation and restore visual acuity. However, transient ocular hypertension was managed successfully with topical therapy [5,6].

## Conclusion

This case highlights the importance of considering leptospirosis in the differential diagnosis of papillitis and retinal vasculitis, even in the absence of fever, jaundice, or clear epidemiological data. In pediatric patients—where such presentations are even rarer—maintaining a high degree of clinical suspicion is essential to avoid diagnostic delays and permanent visual complications. Moreover, post-uveitis complications (e.g., ocular hypertension or cataract) may have more severe consequences in children than in adults, underscoring the need for close follow-up.

Lastly, it should be remembered that a negative PCR result does not rule out the diagnosis, since during the immune-mediated phase, the pathogen has already been cleared from the eye, and the immune response perpetuates the tissue damage. Therefore, the combination of clinical history, characteristic ophthalmological findings, and compatible serology remains the most reliable approach for diagnosing ocular leptospirosis in this phase.
